# Myco-synthesized copper oxide nanoparticles as a sustainable bionanofungicide for managing *Fusarium falciforme* and enhancing potato productivity

**DOI:** 10.1038/s41598-026-52727-w

**Published:** 2026-05-25

**Authors:** Reham U. Ahmed, Alaa M. Abou-Zeid, Ahmed I.S. Ahmed, Khalil M. Saad-Alla

**Affiliations:** 1 Botany and Microbiology Department, Faculty of Science, Tanta University, Tanta, 31527 Egypt; 2 Plant Pathology Unit, Plant Protection Department, Desert Research Center, Cairo, Egypt

**Keywords:** Green nanotechnology, Bionanofungicide, Rhizosphere fungi, Sustainable agriculture, Plant growth promotion, Biochemistry, Biological techniques, Biotechnology, Microbiology, Plant sciences

## Abstract

Developing sustainable alternatives to chemical fungicides is imperative for sustainable agriculture. This study establishes a green nanotechnology approach by harnessing the biocatalytic potential of indigenous rhizosphere fungi to synthesize copper oxide nanoparticles (CuONPs). A consortium of soil-borne fungi, including *Mucor circinelloides*, was isolated from agricultural soils in Tanta, Egypt, and employed in the myco-synthesis of CuONPs. Comprehensive characterization confirmed the formation of crystalline, spherical nanoparticles (average size: 10.7 nm). The nanoparticles exhibited notable, dose-dependent antifungal activity in vitro, suppressing mycelial growth of the potato pathogen *Fusarium falciforme* by 33.95%, significantly outperforming bulk copper sulfate. Based on preliminary dose–response trials, 200 mg L^− 1^ was selected as the optimal concentration balancing antifungal efficacy and absence of phytotoxicity. In a greenhouse trial, foliar application of myco-synthesized CuONPs (200 mg L^− 1^) to *F. falciforme*-infected potato plants mitigated disease-induced growth inhibition, restored photosynthetic pigment levels, and rebalanced antioxidant enzyme systems, primarily enhancing catalase activity. Significantly, CuONPs supported protein homeostasis under stress and, most importantly, boosted tuber yield by up to 40% in healthy plants and appeared to restore productivity in infected ones. In contrast, the commercial fungicide showed phytotoxic effects on tuber initiation. This work establishes myco-synthesized CuONPs as a novel, dual-action agent for plant protection and growth promotion, offering a sustainable and effective strategy for integrated disease management.

## Introduction

Potato (*Solanum tuberosum* L.), as a member of the family Solanaceae, is a vital tuber crop and a global food staple, ranking fourth in consumption after wheat, rice, and maize^1^. According to the most recent FAOSTAT data (released in early 2026), global potato production reached a record high of approximately 390.4 million tons in 2024^2^. Egypt is a significant player, ranking as the sixteenth largest global producer, the leading producer in Africa, and the fifth largest global exporter with annual exports of 637,000 tons^3^. Nutritionally, the potato tuber is composed of roughly 80% water and 20% dry matter. It is a primary source of carbohydrates, containing 60–80% starch, and provides high-quality protein, dietary fiber, vitamins (such as vitamin C and B complex), and essential minerals including potassium, iron, and phosphorus^4^. Potatoes also contain nutritionally significant antioxidants, notably phenolic compounds like chlorogenic acid^5^.

Potato faces significant constraints from a complex of abiotic and biotic stressors that collectively compromise both yield and tuber quality. Abiotic stresses, like drought, heat, and nutrient imbalances, directly inhibit physiological processes, severely reducing tuber initiation and bulking, quality defects like chain-tuberization, and tuber dry matter accumulation^6^. Concurrently, a diverse range of biotic threats persistently attack the crop. Among these, fungal pathogens are particularly damaging. The oomycete *Phytophthora infestans*, the causal agent of late blight, remains the most devastating fungal-like disease, capable of destroying entire fields and necessitating intensive fungicide applications^7^. Importantly, soil-borne *Fusarium* spp. (including *F. falciforme*) causes dry rot, directly disfiguring tubers and reducing marketable yield^8^. While other pathogens, such as bacteria, viruses, and nematodes, also contribute to yield losses, the present study focuses specifically on *F. falciforme* as a target fungal pathogen. This complex interplay between environmental and biological stressors presents a major challenge to sustainable potato production, driving the need for integrated management strategies that include resilient cultivars, precise irrigation, and targeted pest and disease control to safeguard this essential crop.

*Fusarium* dry rot, caused by a complex of *Fusarium* species, has become a predominant fungal disease of potato, leading to significant pre- and postharvest losses globally. The disease manifests as *Fusarium* wilt in the field and, more devastatingly, as a dry rot of tubers during storage, where it can propagate from infected to healthy tubers^9^. Quantitative losses in storage can range from 25% to 60% of the yield. Qualitatively, the disease degrades the tuber’s nutritional composition, adversely affecting starch-related components like amylose and amylopectin, as well as soluble sugar content^10^. The pathogen complex is diverse, with over 13 species implicated worldwide, including *F. oxysporum*,* F. solani*,* F. culmorum*, and *F. sambucinum*, with species prevalence varying by region and cultivar^11^.

Current control primarily relies on synthetic fungicides such as thiabendazole. However, the emergence of fungicide-resistant *Fusarium* strains, coupled with concerns over environmental contamination and chemical residues, has rendered this approach unsustainable^11^. Consequently, research is pivoting towards eco-friendly alternatives. Among the most promising are biological control agents and the application of metallic nanoparticles, which have demonstrated effective antifungal activity and offer a potential path for integrated disease management. The pursuit of sustainable plant disease management has positioned nanotechnology as a pivotal field, with the biological synthesis of nanoparticles offering an eco-friendly alternative to chemical methods. This green synthesis, particularly using fungi, leverages their high metal tolerance, extracellular enzyme secretion, and scalability, resulting in morphologically reproducible and stable nanoparticles without toxic byproducts^12^.

Among various nanomaterials, copper oxide nanoparticles (CuONPs) have garnered significant attention due to their effective and broad-spectrum antimicrobial properties. Copper is also an essential plant micronutrient, acting as a cofactor for critical enzymes involved in photosynthesis, respiration, and oxidative stress response^13^. This dual role as both a nutrient and a protectant makes CuONPs particularly advantageous for agricultural applications^14^. Recent research consistently demonstrated the efficacy of biologically synthesized CuONPs against a wide range of destructive phytopathogenic fungi. For instance, CuONPs biosynthesized using *Trichoderma harzianum* exhibited significant antifungal activity against *Pyricularia oryzae*, the causative agent of wheat blast disease, reducing mycelial growth by up to 74% and disease severity by 95% in greenhouse trials^15^. Similarly, green-synthesized CuONPs using *Trichoderma asperellum* and *Trichoderma ghanense* demonstrated potent inhibitory effects against *Botrytis cinerea*, achieving 100% mycelial growth inhibition at 160 µg mL^− 1^ and outperforming commercial copper-based fungicides^16^. The antifungal action of CuONPs is dose-dependent and is primarily attributed to the generation of reactive oxygen species (ROS), disruption of cell membrane integrity, interference with essential cellular enzymes, and induction of apoptosis in fungal cells^14,17^.

Despite the growing body of research on myco-synthesized CuONPs, several critical gaps remain unaddressed. Most existing studies have focused exclusively on in vitro antifungal activity or single physiological parameters, with limited translation to whole-plant greenhouse trials. Furthermore, no study to date has comprehensively evaluated the dual role of myco-synthesized CuONPs as both a biopesticide against *Fusarium* dry rot and a biofertilizer for growth promotion in a single pathosystem. Specifically, the following gaps are addressed in the present work: (1) the use of *Fusarium falciforme* as a target pathogen for potato dry rot has not been previously investigated with CuONPs; (2) the combined assessment of pathogen suppression, photosynthetic performance, osmolyte balance, antioxidant enzyme modulation, and tuber yield under greenhouse conditions represents a holistic approach absent from prior literature; and (3) a direct comparative evaluation of myco-synthesized CuONPs against both bulk CuSO_4_ and a commercial fungicide (Maxim) under identical experimental conditions has not been reported. Therefore, the present study advances beyond existing literature by providing an integrated, multi-parameter evaluation of myco-synthesized CuONPs from fungal synthesis to field-relevant application, establishing their potential as a dual-action nano-agrochemical for sustainable potato production.

Thereby, the green synthesis of CuONPs presents a promising, sustainable strategy for fungal disease control. Their potent, broad-spectrum antifungal activity, coupled with their nutritional benefit to plants, positions them as a viable component for integrated pest management systems, reducing reliance on conventional chemical fungicides. This study aims to establish a sustainable platform for plant disease management by innovating the green synthesis of CuONPs using soil-borne fungi. We will systematically evaluate the efficacy of these bio-fabricated CuONPs in controlling *Fusarium falciforme* and mitigating potato dry rot. Furthermore, the investigation extends to their role as a nano-spraying agent, assessing their ability to enhance plant growth, photosynthetic pigment content, antioxidant status, and tuber yield criteria in infected potato plants.

## Materials and methods

### Isolation and purification of rhizosphere fungi from agricultural soils

A fungal consortium was isolated from the rhizosphere of vegetable crops in Tanta, Egypt, to establish a collection of indigenous soil microbiota. Fungal propagules were isolated from a 1 g soil aliquot using suspension and serial dilution in sterile distilled water, a standard method for quantifying and reducing microbial load^18^. Subsequently, a 0.5 mL aliquot of the diluted supernatant was aseptically spread onto a selective culture medium of Potato Dextrose Agar (PDA), which was amended with chloramphenicol to create a bacterially suppressive environment conducive to fungal growth.

Following incubation at 30 °C for a period of 3–5 days, a taxonomically diverse array of fungal colonies emerged. The isolated mycobiota included multiple genera of significant ecological and phytopathological relevance. The identified fungal genera included *Aspergillus* spp., *Fusarium* spp., *Alternaria* spp., *Chaetomium* spp., *Cladosporium* spp., *Rhizopus* spp., and *Mucor* spp. Each morphologically distinct colony was purified through sequential subculturing on fresh PDA plates to obtain genetically homogeneous, axenic cultures. Final identification of these purified isolates was conducted through a comprehensive morphological analysis. This involved examining both macroscopic (colony morphology, pigmentation, growth rate) and microscopic (hyphal structure, conidiophore architecture, spore morphology) characteristics based on authoritative taxonomic manuals and mycological keys^19–21^.

### Myco-synthesis of copper oxide nanoparticles (CuONPs)

For the biological synthesis of CuONPs, each previously isolated fungal strain was cultivated under controlled conditions to generate bioactive filtrates and biomass. A 2 mL aliquot of a standardized spore suspension was used to inoculate 250 mL Erlenmeyer flasks containing 100 mL of sterile malt extract, glucose, yeast extract, and peptone (MGYP) broth. The cultures were incubated at 30 °C for 5 days on an orbital shaker at 150 rpm to facilitate uniform mycelial growth and metabolite production. Post-incubation, the mycelial biomass was separated from the culture broth via vacuum filtration through Whatman No. 1 filter paper. The harvested fresh biomass was weighed, thoroughly washed with sterile deionized water to remove residual media components, and reserved for synthesis. The cell-free culture filtrate from each isolate was collected as the primary source of extracellular fungal exudates^22,23^.

Two distinct extracellular synthesis protocols utilizing fungal metabolites were implemented. The first approach, exudate-mediated synthesis, was conducted by directly combining 10 mL of the cell-free culture filtrate, containing secreted fungal metabolites, with 90 mL of a 10 mM aqueous copper sulfate (CuSO_4_) solution (1:9 v/v). In the second, biomass extract-mediated synthesis, 10 g of washed fresh mycelial biomass was first subjected to aqueous extraction by suspension in 100 mL of sterile deionized water under agitation at room temperature for 48 h. The resulting extract, enriched with cell-wall-associated biomolecules and exudates, was filtered and then mixed with the CuSO_4_ solution (1:9 v/v). In both methods, the reaction mixtures were incubated at 28 °C for 72 h on an orbital shaker at 150 rpm. All syntheses were performed in triplicate to ensure reproducibility. Following this biosynthesis phase, the formation of CuONPs was visually confirmed by the appearance of a characteristic black precipitate. This precipitate was then separated and subjected to an identical purification protocol through sequential washing with deionized water and absolute ethanol to remove biological residues, followed by oven-drying at 80 °C for 24 h to yield a stable powder for subsequent characterization.

### Characterization of the myco-synthesized CuONPs

The initial detection of CuONPs was performed by visual observation. A color change in the reaction mixture to a dark-brown or black hue was considered evidence of CuONPs formation^24^. The obtained precipitate was then characterized using X-ray diffraction (XRD), Fourier-transform infrared spectroscopy (FT-IR), transmission electron microscopy (TEM), and zeta potential analysis.

XRD analysis was performed using a Philips PW1710 instrument equipped with Cu-Kα radiation (λ = 1.54060 Å). This technique was used to determine the crystalline nature of the prepared samples and to investigate interactions between functional groups. Samples were analyzed with a scanning speed of 0.02° min^− 1^ over a 2θ range of 2° to 80°. Magnetic hysteresis (M-H, Lakeshore 7400) curves for samples were measured at room temperature under an applied magnetic field ranging from − 8 to 8 kOe.

FT-IR was conducted using a Shimadzu FTIR-8101 A spectrometer. Spectra were recorded in the range of 4000–400 cm^− 1^ using potassium bromide (KBr) discs, with 64 scans and a resolution of 4 cm^− 1^ per sample.

The size distribution and surface charge of the biosynthesized NPs were determined by dynamic light scattering (DLS) using a Zetasizer Nano ZS instrument (Malvern Instruments, UK). Electrochemical measurements were performed using computerized analyzers (Models 263 A and 394-PAR, Princeton Applied Research) with accompanying 270/250-PAR software. A three-electrode sensor assembly (303 A-PAR) was employed, consisting of a graphite paste working electrode, an Ag/AgCl/KCl(s) reference electrode, and a platinum wire auxiliary electrode, all integrated within a micro-electrochemical cell.

The morphological structure and particle size of the NPs were investigated using high-resolution transmission electron microscopy (HR-TEM) on a JEM-2100 instrument (JEOL). Samples were prepared by depositing drops of the dispersed material onto a grid from a mixture of ethanol and double-distilled water (DDW).

### Isolation and molecular identification of *Fusarium falciforme*

The fungal pathogen was isolated from symptomatic potato tubers. Diseased tissue from lesion margins was surface-sterilized with 70% ethanol and plated onto Potato Dextrose Agar (PDA), followed by incubation at 30 °C for 3–5 days and subsequent purification via subculturing^25^. For molecular identification, genomic DNA was extracted from a pure culture using a commercial silica-membrane kit (Solg™ Genomic DNA Prep Kit, SolGent, South Korea). The Internal Transcribed Spacer (ITS) region of ribosomal DNA was amplified via PCR with universal primers ITS1 and ITS4^26^ using a Bio-Rad T100 Thermal cycler. The resulting amplicons were purified, sequenced bidirectionally (SolGent Company, South Korea) using an ABI 3730xl DNA Analyzer and assembled. The consensus sequence was analyzed using BLAST against the NCBI GenBank database, and phylogenetic relationships were inferred using Clustal W alignment in MegAlign software (DNASTAR 15.0)^27^, confirming the isolate as *Fusarium falciforme*.

### In vitro antifungal activity assay of CuONPs

The antifungal efficacy of the synthesized CuONPs against *Fusarium falciforme* was evaluated using the poisoned food technique. Potato dextrose agar (PDA) was amended with CuONPs at a concentration of 200 mg L^− 1^ before being poured into plates. This concentration was selected based on preliminary dose–response assays (50–500 mg L^− 1^), which showed that 200 mg L^− 1^ was the minimum concentration achieving consistent antifungal activity (> 30% growth inhibition) without causing particle aggregation or excessive toxicity that could confound interpretation of fungal growth. A 5-mm mycelial plug from an actively growing fungal culture was aseptically transferred to the center of each plate. Following incubation at 30 °C for seven days in a Memmert INE400 microbiological incubator, the radial mycelial growth was measured. This protocol was performed in triplicate and included control plates of unamended PDA, as well as plates supplemented with an equivalent concentration of bulk copper sulfate and a commercial antifungal agent for comparative analysis^28^.

### Greenhouse experiment

#### Inoculum preparation and experimental setup

A virulent inoculum of *Fusarium falciforme* was prepared using an established grain-based method. The fungus was first isolated from diseased potato tissue (lesion margins) following the established grain-based method. Sterile 250 mL flasks were filled to half capacity with a sterile substrate mixture composed of sorghum grains, sand, and distilled water in a 2:1:1 (w/w/v) ratio as described by^29^. Following autoclaving (Tomy SX-700 autoclave), each flask was inoculated with two 5-mm mycelial discs obtained from the margin of a 7-day-old *F. falciforme* culture grown on PDA at 30 °C. The inoculated flasks were then incubated at 30 °C for 7 days with periodic shaking to ensure uniform colonization, resulting in a heavily sporulated substrate where all sorghum grains were fully enveloped by fungal mycelia^30^. Concurrently, plastic pots (35 cm diameter, 30 cm depth) were surface sterilized with a 5% formalin solution and air-dried. Each pot was filled with 8 kg of a pre-sterilized soil mixture (clay and sand, 2:1 w/w). To establish the disease pressure, the soil was lightly watered, and 10 g of the fully colonized grain inoculum was thoroughly incorporated into the topsoil layer of each designated pot. These infested pots were then maintained for five days under greenhouse conditions to allow for initial pathogen establishment before tuber sowing.

#### Treatment application and experimental design

The experiment utilized potato tubers (*Solanum tuberosum* cv. Spunta) obtained from the Ministry of Agriculture and Land Reclamation (MALR), Egypt, to evaluate the protective efficacy of various treatments against dry rot. Before planting, tubers were topically sprayed using a handheld sprayer (Chapin 20000) until they run-off with one of four treatment solutions: (a) 200 mg L^− 1^ myco-synthesized CuONPs, (b) 200 mg L^− 1^ bulk CuSO_4_ (procured from Sigma-Aldrich, Egypt), (c) 0.1% commercial fungicide (Maxim, Syngenta, Egypt), or (d) sterile distilled water serving as an untreated negative control. Immediately after treatment, a single tuber was sown in the center of each pre-infested pot. The experimental design included control groups: pots infested with *F. falciforme* and sown with water-treated tubers served as the positive (diseased) control, while non-infested pots sown with water-treated tubers constituted the negative (healthy) control. The entire experiment was arranged in a completely randomized design (CRD) with three biological replicates per treatment, conducted in a greenhouse at the Faculty of Science, Tanta University, from November 2023 to March 2024.

#### Crop management, disease assessment, and pathogen re-isolation

Throughout the 100-day growing season, plants were maintained under natural light and temperature regimes within the greenhouse and irrigated with tap water as needed. To sustain the protective effect, a foliar application regimen was implemented wherein each plant was sprayed with 25 mL of its respective treatment solution at a concentration of 200 mg L^− 1^ (or the equivalent for the fungicide and water controls) at 14-day intervals using the same handheld sprayer. Plant growth was monitored weekly, and the onset and progression of dry rot symptoms were meticulously recorded. Upon harvest, tubers were air-dried at room temperature for three days before final disease assessment. To conclude, *F. falciforme* as the causative agent, symptomatic tissue from control plants was surface-sterilized and plated onto PDA. The re-isolated fungus was morphologically identified, thereby verifying the disease etiology.

### Assessment of plant growth and photosynthetic pigments

The shoot length (cm) was measured from the soil surface at 25 days post-planting. Concurrently, leaf area (mm^2^) was measured for the second fully expanded leaf from three plants per treatment using a BenQ 500B desktop scanner. Digital leaf images were analyzed with Scion imaging software (v. 4.0.2) to calculate surface area. Following measurement, leaf samples were oven-dried (Memmert UF 110) at 60 °C to constant weight, pulverized with an electric mixer (Black+Decker MX3200B), sieved through a 0.2 mm mesh (Endecotts No. 70), and stored in paper bags for subsequent biochemical analysis.

Photosynthetic pigment composition was assessed using established spectrophotometric methods. Fresh leaf tissue (0.1 g) was homogenized in 10 mL of ice-cold 80% acetone using a pre-chilled mortar and pestle. The homogenate was centrifuged using an Eppendorf 5804R centrifuge at 6000 rpm for 15 min, and the absorbance was measured at 663, 644, and 452.5 nm using a UV-Vis spectrophotometer (Shimadzu UV-1800). Chlorophylls a and b, and carotenoid concentrations (mg g^− 1^ FM) were calculated according to^31^.

### Analysis of osmoprotectant metabolites

Total soluble carbohydrates and proteins were extracted in borate buffer (pH 8.5). For quantification of total soluble carbohydrates (TSC), 0.1 mL of the supernatant was reacted with 0.1 mL of 5% aqueous phenol (w/v) and 1 mL of concentrated sulfuric acid. The mixture was vortexed with a benchtop vortex mixer (Stuart SA3), incubated at 60 °C for 20 min in a water bath, cooled, and its absorbance was measured at 490 nm. TSC concentration (mg g^− 1^ DM) was determined using a glucose standard curve prepared according to the phenol‑sulfuric acid method^32^.

The soluble protein (TSP) concentration in the borate extracts was determined via the Bradford assay^33^. Aliquot of 0.1 mL extract was mixed with 2.9 mL of Coomassie Brilliant Blue G‑250 reagent, and absorbance was measured at 595 nm. Quantification of TSP (mg g^− 1^ DM) was performed using a standard curve generated with bovine serum albumin (BSA).

### Quantification of non-enzymatic antioxidants

An ethanolic extract was prepared for antioxidant analysis by incubating 0.1 g of dried, powdered leaf material in 10 mL of 80% ethanol for 24 h at room temperature, followed by filtration. Total phenolic content was determined using the Folin-Ciocalteu method^34^. Briefly, 0.1 mL extract was reacted with 0.1 mL Folin-Ciocalteu reagent, 1 mL 20% Na_2_CO_3_, and 1.8 mL distilled water. After a one-hour incubation at room temperature, absorbance was measured at 650 nm. Phenols quantification was performed using a standard curve prepared with gallic acid, and results were expressed as milligrams of gallic acid equivalents per gram of dry mass (mg GAE g^− 1^ DM).

Total flavonoid content was assessed according to^35^. A 0.5 mL aliquot of the extract was mixed with 0.1 mL of 10% AlCl_3_, 0.1 mL of 1 M K-acetate, and 1.5 mL of 95% ethanol. The mixture was incubated for 30 min, and absorbance was recorded at 417 nm. A quercetin standard curve was used for flavonoid quantification as milligrams of quercetin equivalents per gram of dry mass (mg QE g^− 1^ DM).

### Assay of antioxidant enzyme activities

Freshly harvested potato leaves (0.5 g) were homogenized at 4 °C in 8 mL of 0.1 M K-phosphate buffer (pH 7.0). The homogenates were centrifuged at 6,000 rpm for 20 min at 4 °C, and the resulting supernatant was used immediately for spectrophotometric enzyme assays^36^.

Catalase (CAT) activity was measured by monitoring H_2_O_2_ decomposition at 240 nm^36^. The reaction mixture (3 mL) consisted of 50 mM K-phosphate buffer (pH 7.0) and 15 mM H_2_O_2_. The reaction was initiated with 0.1 mL enzyme extract, and the decrease in absorbance was used to calculate CAT activity (µmol g^− 1^ FM min^− 1^) using an extinction coefficient of 40 mM^− 1^ cm^− 1^.

Peroxidase (POD) activity was determined by guaiacol oxidation^36^. A 3 mL reaction mixture containing 100 mM K-phosphate buffer (pH 5.8), 7.2 mM guaiacol, and 11.8 mM H_2_O_2_ was combined with 0.1 mL enzyme extract. The increase in absorbance at 470 nm was recorded, and POD activity (µmol g^− 1^ FM min^− 1^) was calculated using an extinction coefficient of 26.6 mM^− 1^ cm^− 1^.

Ascorbate peroxidase (APX) activity was assayed according to^37^. The reaction mixture (2 mL) contained 50 mM K-phosphate buffer (pH 7.0), 0.2 mM EDTA, 0.5 mM ascorbic acid, and 0.25 mM H_2_O_2_. The reaction was initiated with 0.1 mL enzyme extract, and the decrease in absorbance at 290 nm was monitored. APX activity (µmol g^− 1^ FM min^− 1^) was calculated using an extinction coefficient of 2.8 mM^− 1^ cm^− 1^.

Polyphenol oxidase (PPO) activity was assayed by monitoring purpurogallin formation^38^. The reaction mixture contained 0.25 mL enzyme extract, 1 mL of 100 mM K-phosphate buffer (pH 6.0), and 0.5 mL of 2 mM pyrogallol. After a 5-min incubation at 25 °C, the reaction was terminated with 0.5 mL of 2.5 N H_2_SO_4_. Absorbance at 420 nm was measured, and PPO activity (µmol g^− 1^ FM min^− 1^) was calculated using 26.4 mM^− 1^ cm^− 1^ as an extinction coefficient.

### Assessment of tuber yield attributes

Key agronomic yield parameters, including the number of tubers per plant and tuber fresh weight (kg plant^− 1^), were determined at harvest according to the methodology described by^39^.

### Statistical analysis

All experimental data are presented as mean ± standard deviation (SD) of three independent biological replicates (*n* = 3). Statistical analysis was performed using CoStat software (v. 6.311, CoHort, Monterey, CA, USA). A one-way analysis of variance (ANOVA) was applied to determine the significance of treatment effects. Where significant differences were detected (*P* < 0.05), means were separated using the Tukey HSD post-hoc test. A probability value of *P* < 0.05 was considered statistically significant for all analyses.

## Results

### Characterization of myco-synthesized copper oxide nanoparticles (CuONPs)

**Fig. 1:**
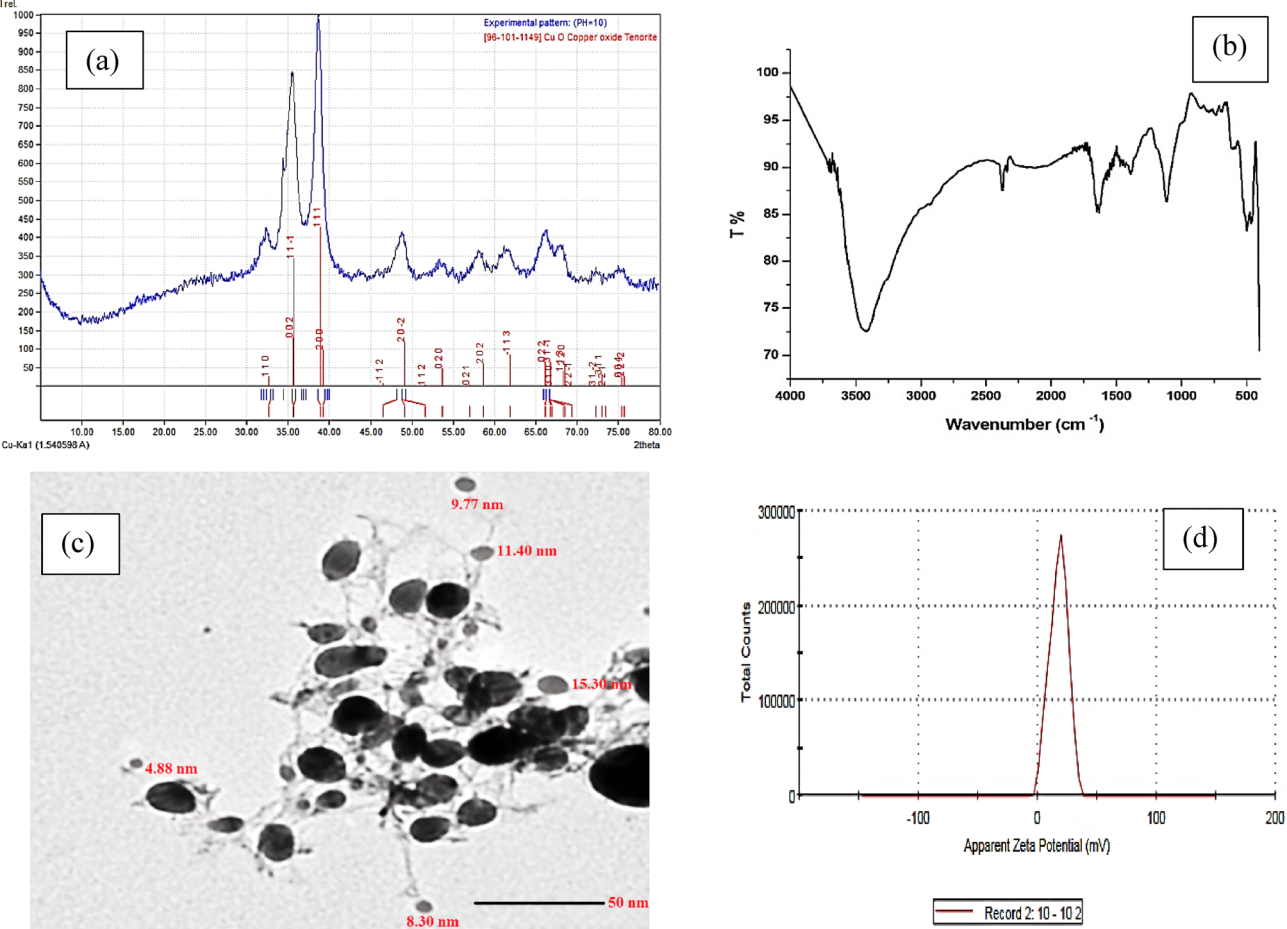
Characterization of myco-synthesized CuONPs: **a** XRD pattern, **b** FT-IR profile, **c** TEM micrograph, and **d** zeta potential.

The structural, functional, and morphological properties of the myco-synthesized copper oxide nanoparticles (CuONPs) were systematically characterized using a multi-technique approach. X-ray diffraction (XRD) analysis (Fig. 1a) confirmed the crystalline nature and phase of the biosynthesized material. The diffraction pattern exhibited characteristic peaks at 2θ values of 32.85°, 35.49°, 38.65°, 48.80°, 61.65°, 66.63°, and 68.10°, corresponding to the (110), (11 − 1), (111), (20 − 2), (−113), (31 − 1), and (220) crystallographic planes, respectively. The complete pattern, including the absence of impurity peaks, is consistent with the monoclinic tenorite phase of copper oxide (JCPDS Card No. 96–101−1149). The average crystallite size, calculated using the Debye-Scherrer equation, was 10.7 nm.

Fourier-transform infrared (FTIR) spectroscopy (Fig. 1b) identified the functional biomolecules involved in nanoparticle synthesis and stabilization. A broad absorption band in the range of 3376–3412 cm^− 1^ is attributed to O–H stretching vibrations of phenols, alcohols, or adsorbed water. A distinct band at 2371 cm^− 1^ corresponds to O–C = O stretching, while the band at 1629 cm^− 1^ is indicative of the amide I (C = O stretching) region of fungal proteins or aromatic C = C stretching. Absorptions between 1105 and 1117 cm⁻¹ suggest the presence of C–O–C/C–O vibrations from polysaccharides or residual sulfate ions. The vibrational bands in the fingerprint region (606–426 cm^− 1^) are characteristic of the Cu–O stretching modes, confirming the formation of copper oxide.

Transmission electron microscope (TEM) micrographs revealed that the nanoparticles synthesized using *Mucor* sp. mycelial extract at pH 10 were well-dispersed and predominantly hemispherical in morphology (Fig. 1c). The particle size distribution ranged from 4 to 20 nm, with an average size consistent with the XRD-derived crystallite dimension.

The colloidal stability of the synthesized CuONPs was assessed via zeta potential measurement. The nanoparticles exhibited a zeta potential value of + 18.06 mV, indicating a moderate positive surface charge and suggesting reasonable electrostatic stabilization in suspension.

### Molecular identification of the pathogenic fungal strain

**Fig. 2:**
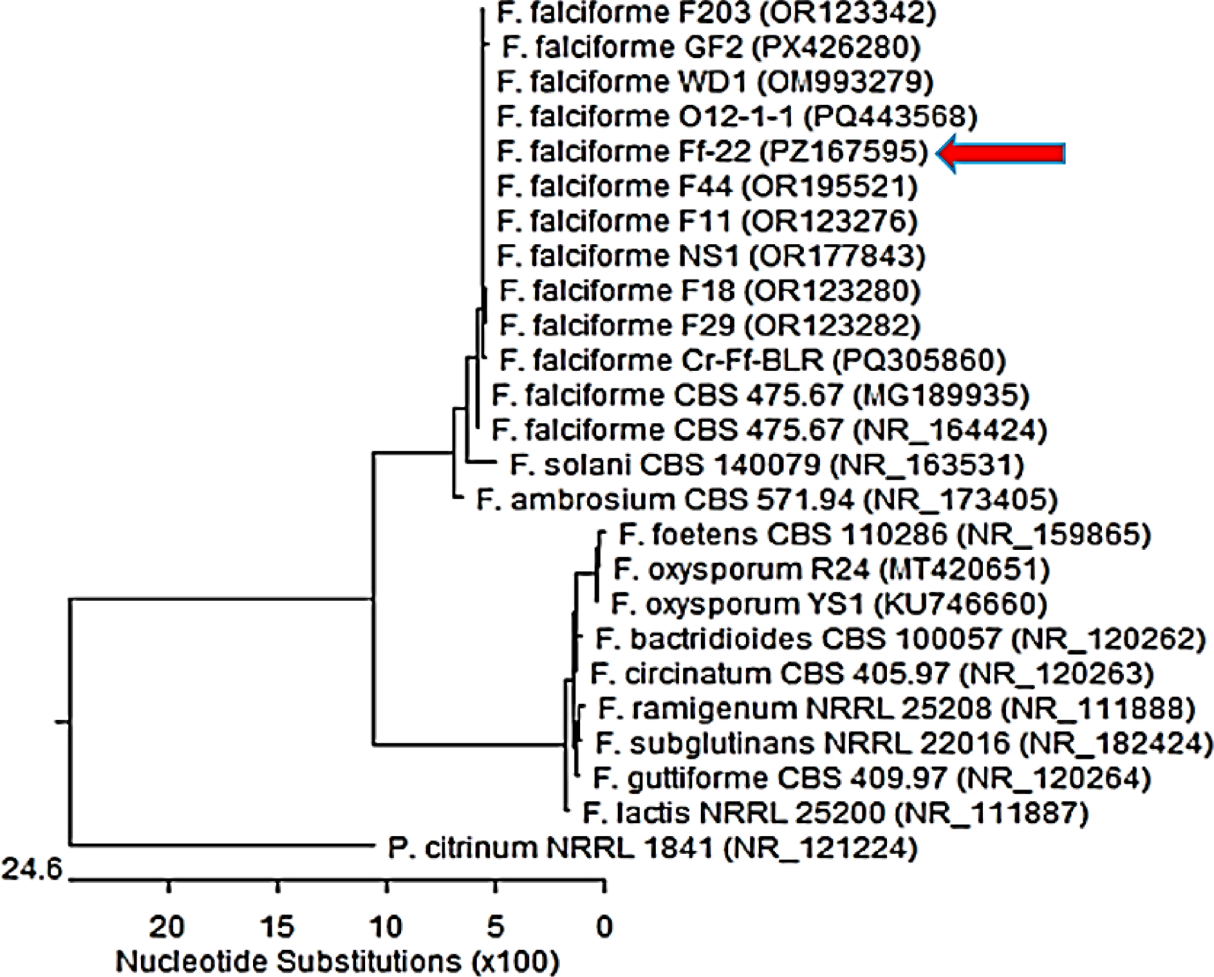
Neighbor-joining phylogenetic tree illustrating the identification of *Fusarium falciforme* Ff-22 (PZ167595) based on ITS rDNA sequence analysis.

The successful molecular identification of the target fungal pathogen, via the sequencing and phylogenetic analysis of the Internal Transcribed Spacer (ITS) region, provides a robust and specific foundation for this study. The ITS sequence generated was submitted to the NCBI GenBank database, and comparative analysis using the Basic Local Alignment Search Tool (BLAST) revealed 99–100% identity with *Fusarium falciforme* Ff-22 (PZ167595) (Fig. 2). This high-percentage identity, coupled with a low E-value, provided strong confirmation at the species level. This precise identification is critical, as *F. falciforme* is a recognized soil-borne pathogen associated with wilt and root rot diseases in numerous crops.

### In vitro antifungal activity against *Fusarium falciforme*

**Fig. 3:**
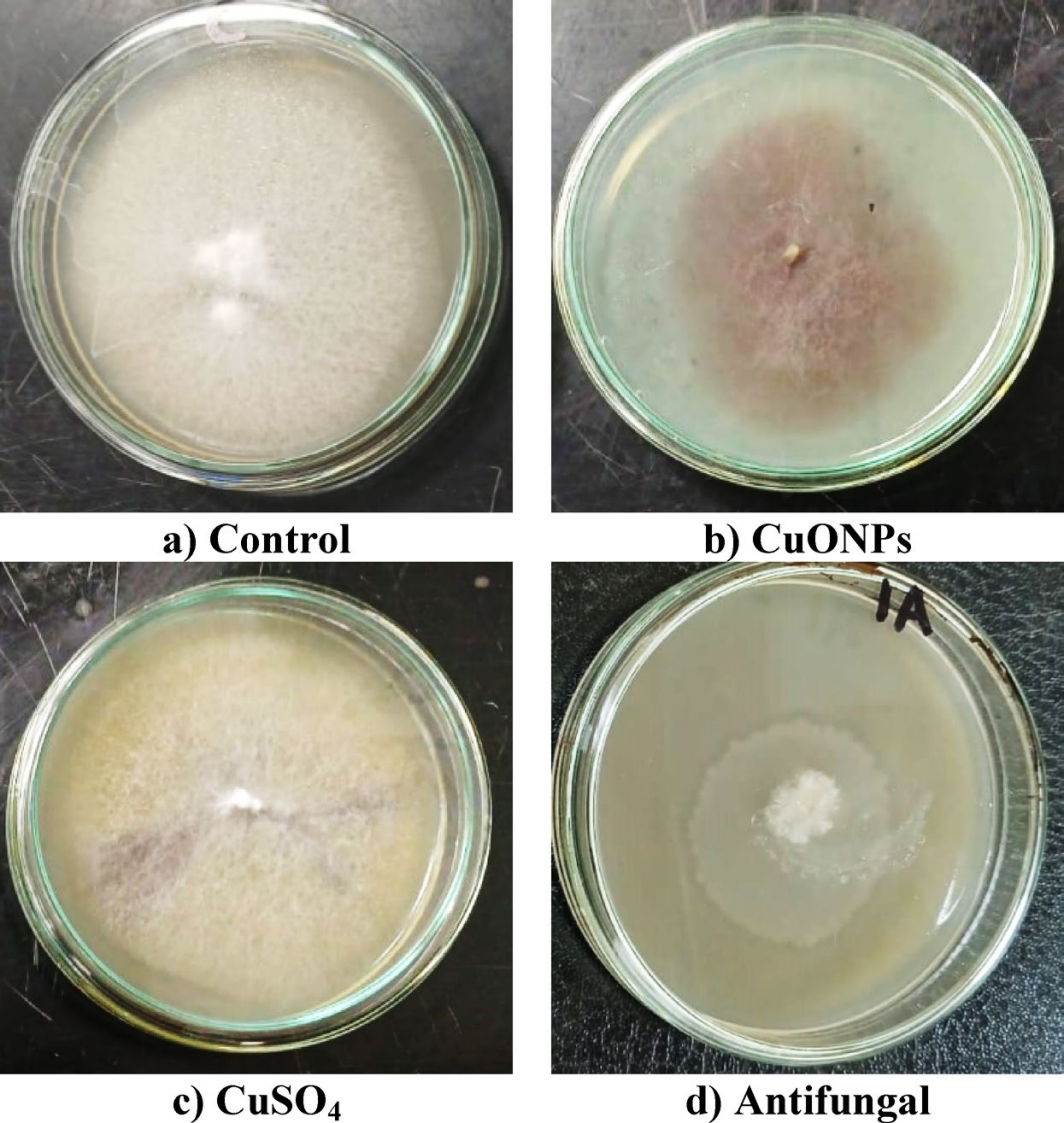
Comparative antifungal activity against *F. falciforme* on PDA plates: **a** unamended control, **b** myco-synthesized CuONPs, **c** bulk CuSO_4_, and **d** commercial antifungal (maxim).

The comparative in vitro antifungal efficacy of the myco-synthesized CuONPs, bulk copper sulfate (CuSO_4_), and the commercial fungicide (Maxim) were evaluated against the phytopathogen *F. falciforme* using the poisoned-food technique on solid PDA medium (Table 1; Fig. 3). The results showed that the myco-CuONPs exhibited potent, dose-dependent antifungal activity. At the highest tested concentration (200 mg L^− 1,^ which was selected based on preliminary dose–response assays at 50–500 mg L^− 1^ concentrations achieving the optimal balance between efficacy and particle stability), myco-CuONPs suppressed radial mycelial growth by 33.95%, reducing it from 8.93 cm (control) to 5.92 cm. In contrast, an equimolar concentration of bulk CuSO_4_ showed negligible efficacy, with only a 2.24% inhibition (8.93 cm to 8.73 cm). The commercial fungicide demonstrated the strongest inhibition at 46.64%. Notably, both copper treatments induced violet pigmentation in the fungal colony, a potential indicator of metal-induced stress or a specific phenotypic response.

**Table 1:** In vitro antifungal efficacy of treatments against *Fusarium falciforme*. Radial mycelial growth (cm) and corresponding inhibition percentage (%) of *F. falciforme* on PDA amended with myco-synthesized CuONPs (200 mg L^1^), bulk CuSO_4_ (200 mg L^− 1^), a commercial antifungal (Maxim, 10 g L^1^), and unamended control. Data represents mean ± SD (*n* = 3).

	Control	CuONPs	CuSO_4_	Antifungal
Growth diameter (cm)	8.93 ± 0.11	5.92 ± 0.26	8.73 ± 0.11	4.767 ± 0.21
Inhibition percent (%)	–	33.95	2.24	46.64

### Results of the pot experiment

#### Growth attributes and photosynthetic pigments

**Fig. 4:**
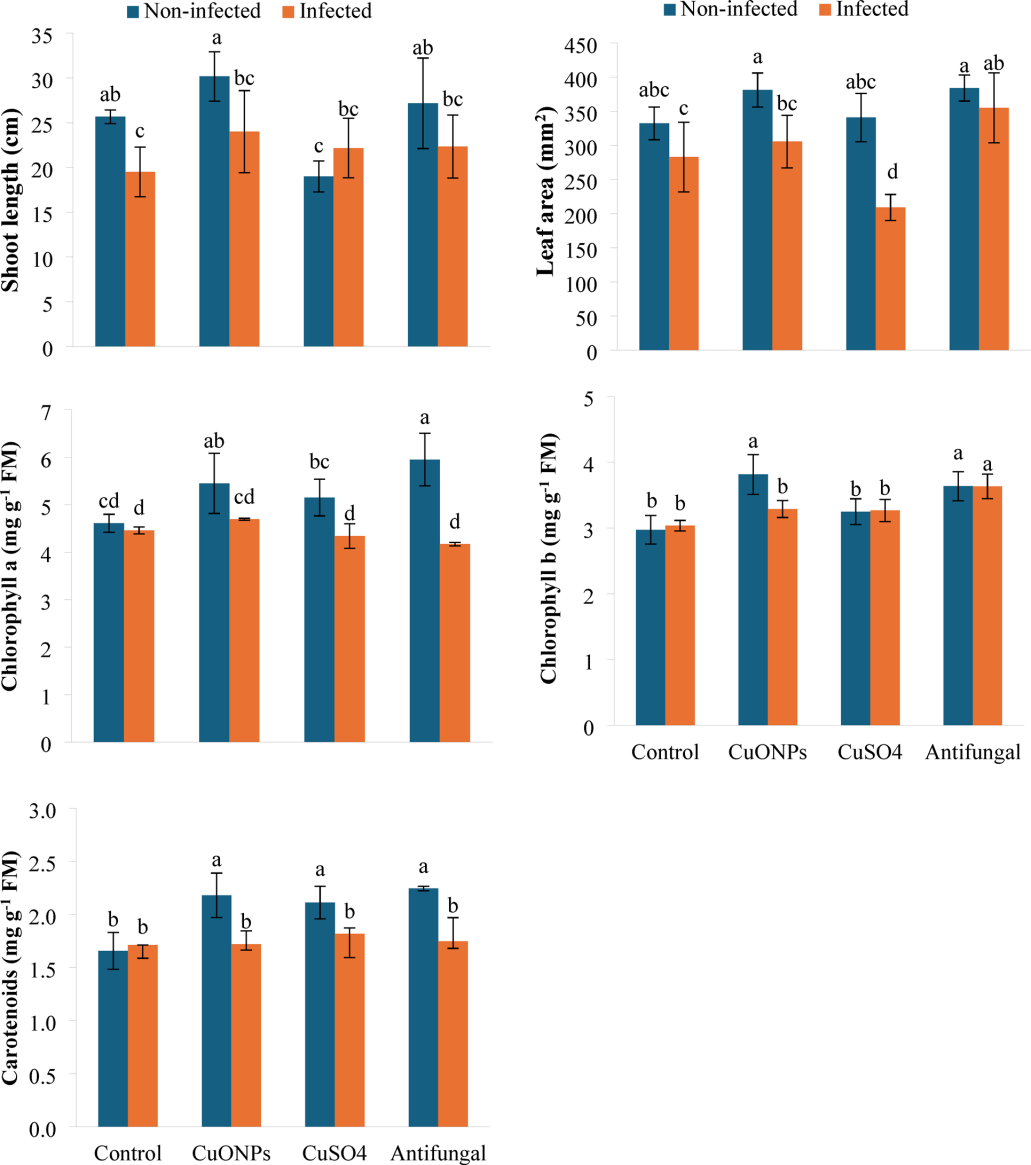
Growth attributes and photosynthetic pigment content in *F. falciforme*-infected potato plants following foliar application of myco-synthesized CuONPs (200 mg L^− 1^). Data are presented as mean ± SD (*n* = 3). Different lowercase letters indicate statistically significant differences (*P* < 0.05) according to one-way ANOVA followed by Tukey HSD post-hoc test.

The foliar application of treatments significantly modulated the growth pattern and photosynthetic pigments of potato plants under *F. falciforme* stress (Fig. 4). Pathogen infection alone caused a marked reduction in shoot length (24.02%) and leaf area (14.86%) compared to healthy controls. Myco-synthesized CuONPs (200 mg L^− 1^) demonstrated a discernible growth-promoting and protective effect. In infected plants, CuONPs not only mitigated the pathogen-induced stunting but stimulated shoot length and leaf area by 23.08 and 8.04%, respectively, relative to the diseased control. Notably, in non-infected plants, CuONPs also enhanced growth (shoot length: +17.53%; leaf area: +14.34%). In contrast, bulk CuSO_4_ exhibited phytotoxicity in healthy plants, inhibiting shoot length by 25.97%, and failed to protect leaf area in infected plants, causing a 26.12% reduction. The commercial antifungal agent showed variable efficacy, promoting growth in infected plants but with a less consistent restorative effect than the nano-formulation. Based on these findings, myco-synthesized CuONPs exhibit a dual function; they act as a biopesticide by suppressing *F. falciforme* infection and mitigating disease symptoms, while also functioning as a biofertilizer by enhancing plant growth parameters (shoot length and leaf area) even in the absence of pathogen infection.

Furthermore, foliar application treatments differentially influenced the photosynthetic pigment profile in potato plants under *F. falciforme* stress (Fig. 4). While infection alone slightly reduced chlorophyll a (−3.23%) and increased chlorophyll b (+ 2.05%) and carotenoids (+ 3.21%), indicating a mild stress response, the applied treatments elicited more significant modulation. Myco-synthesized copper CuONPs consistently acted as a potent photosynthetic enhancer. In non-infected plants, CuONPs substantially increased all pigment concentrations (Chl a: +18.27%; Chl b: +28.30%; carotenoids: +31.56%). Remarkably, in infected plants, CuONPs reversed the pathogen-induced decline in Chl a, inducing a 5.27% increase, while also boosting Chl b (+ 8.44%) and carotenoids (+ 0.8%), relative to the infected control. In contrast, bulk CuSO_4_ and the commercial fungicide exhibited context-dependent effects, generally promoting pigments in healthy plants but showing limited or negative impacts in infected ones, as CuSO_4_ reduced infected-plant Chl a (−2.65%), and the fungicide reduced it further (−6.50%). This photosynthetic enhancement further supports the biofertilizer role of CuONPs.

#### Osmolyte molecules

**Fig. 5:**
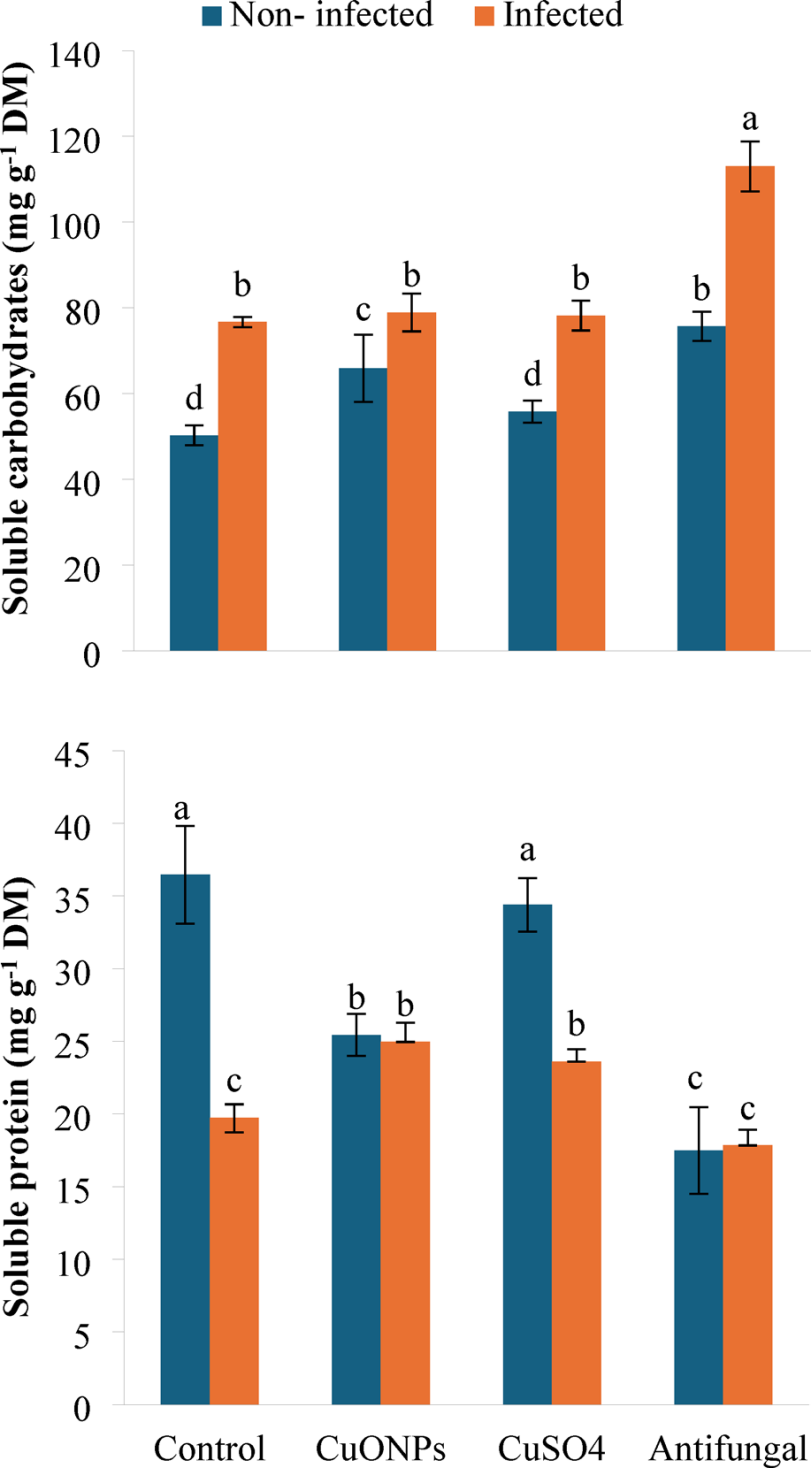
Soluble carbohydrate and protein contents in *F. falciforme*-infected potato plants following foliar application of myco-synthesized CuONPs (200 mg L^− 1^). Data are presented as mean ± SD (*n* = 3). Different lowercase letters indicate statistically significant differences (*P* < 0.05) according to one-way ANOVA followed by Tukey HSD post-hoc test.

The foliar application of treatments in this experiment induced significant and contrasting shifts in primary carbon and nitrogen metabolism, affecting osmolyte molecules (Fig. 5). *F. falciforme* infection alone triggered a substantial metabolic reprogramming, characterized by a 52.55% accumulation of total carbohydrates alongside a 45.87% depletion of protein content, indicative of a stress-induced shift toward osmolyte synthesis at the expense of structural proteins.

Notably, myco-synthesized CuONPs modulated this dysregulated metabolism in infected plants. While slightly elevating carbohydrate levels (+ 2.89%), CuONPs effectively counteracted the pathogen-induced proteolysis, restoring protein content by 26.44% relative to the infected control. This may suggest a role in mitigating stress-related protein degradation. In contrast, bulk CuSO_4_ had a minimal effect on carbohydrates (+ 1.99%) and a moderate positive effect on proteins (+ 19.61%) in infected plants. The commercial antifungal agent induced the highest carbohydrate accumulation in both healthy (+ 50.62%) and infected (+ 47.38%) plants, but significantly suppressed protein levels, particularly in non-infected plants (−52.04%). The ability of CuONPs to restore protein homeostasis under biotic stress reinforces their biopesticide function by alleviating pathogen-induced metabolic damage.

#### Antioxidant metabolites

**Fig. 6:**
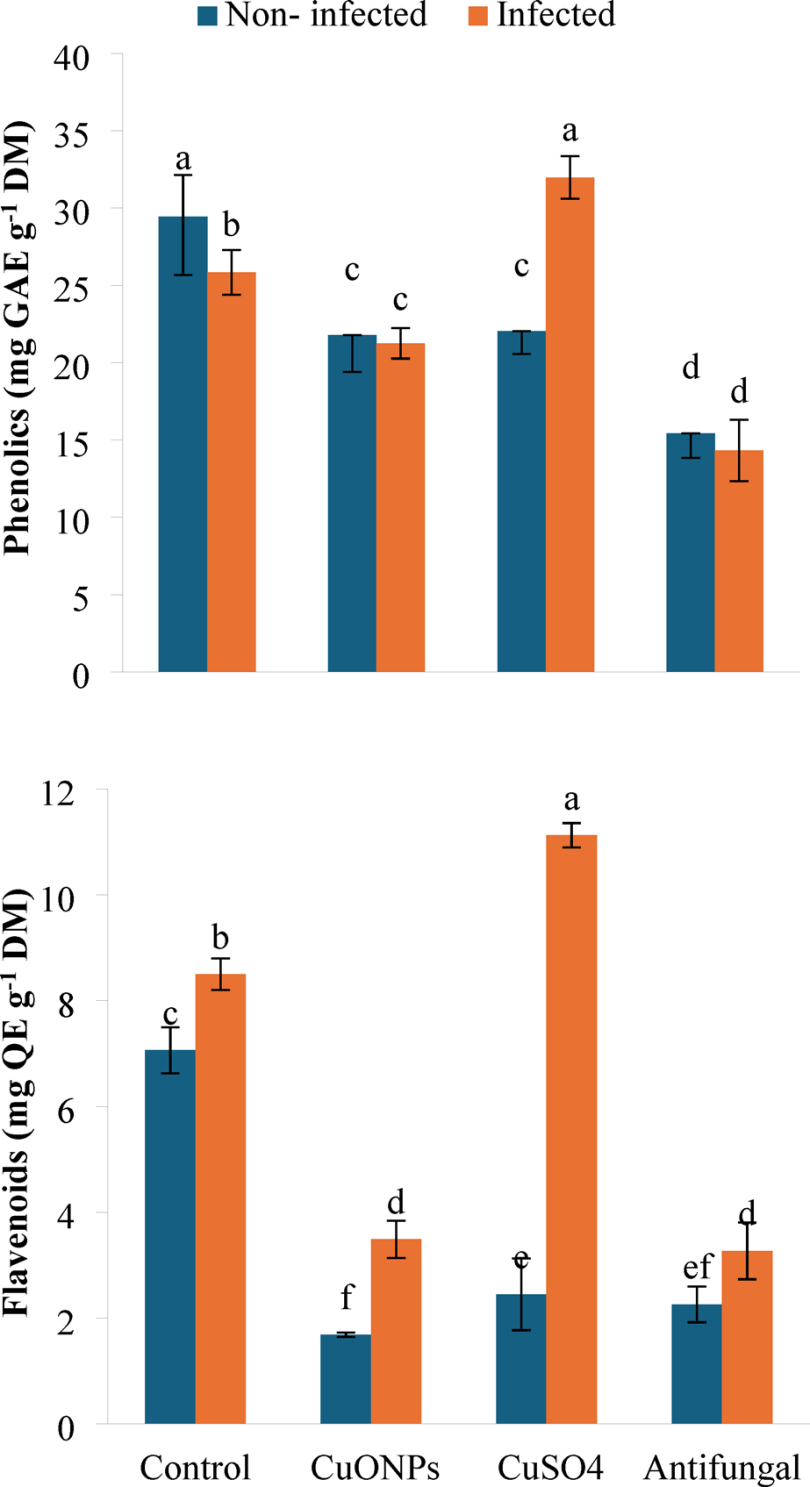
Phenolic and flavonoid contents in *F. falciforme*-infected potato plants following foliar application of myco-synthesized CuONPs (200 mg L^− 1^). Data are presented as mean ± SD (*n* = 3). Different lowercase letters indicate statistically significant differences (*P* < 0.05) according to one-way ANOVA followed by Tukey HSD post-hoc test.

The foliar application of myco-synthesized CuONPs, bulk CuSO_4_, and the commercial antifungal Maxim differentially modulated the pool of non-enzymatic antioxidants in potato plants, with distinct patterns emerging between infected and non-infected treatments (Fig. 6). In healthy plants, all treatments significantly diminished the synthesis of both phenolics (reductions of 26.0, 25.1, and 47.6%, respectively) and flavonoids (reductions of 76.2, 65.3, and 68.0%, respectively) relative to the untreated control.

Infection by *F. falciforme* individually induced a divergent response, slightly decreasing phenolic content (−12.2%) while elevating flavonoids (+ 20.3%). In this infected treatment, CuONPs and the antifungal agent further reduced both phenolic and flavonoid levels. Notably, bulk CuSO_4_ exerted a contrasting effect under pathogen stress, stimulating the accumulation of phenolics (+ 23.8%) and flavonoids (+ 30.9%) compared to the infected control. This suggests that the ionic copper stress from CuSO_4_ may synergize with biotic stress to amplify the synthesis of specific antioxidant compounds, whereas the CuONPs and the fungicide did not elicit this response. These findings highlight treatment and stress-specific reprogramming of phenylpropanoid metabolism, indicating that the induction of these antioxidant pathways is not a common mechanism of action for the tested agents.

#### Antioxidant enzymes

**Fig. 7:**
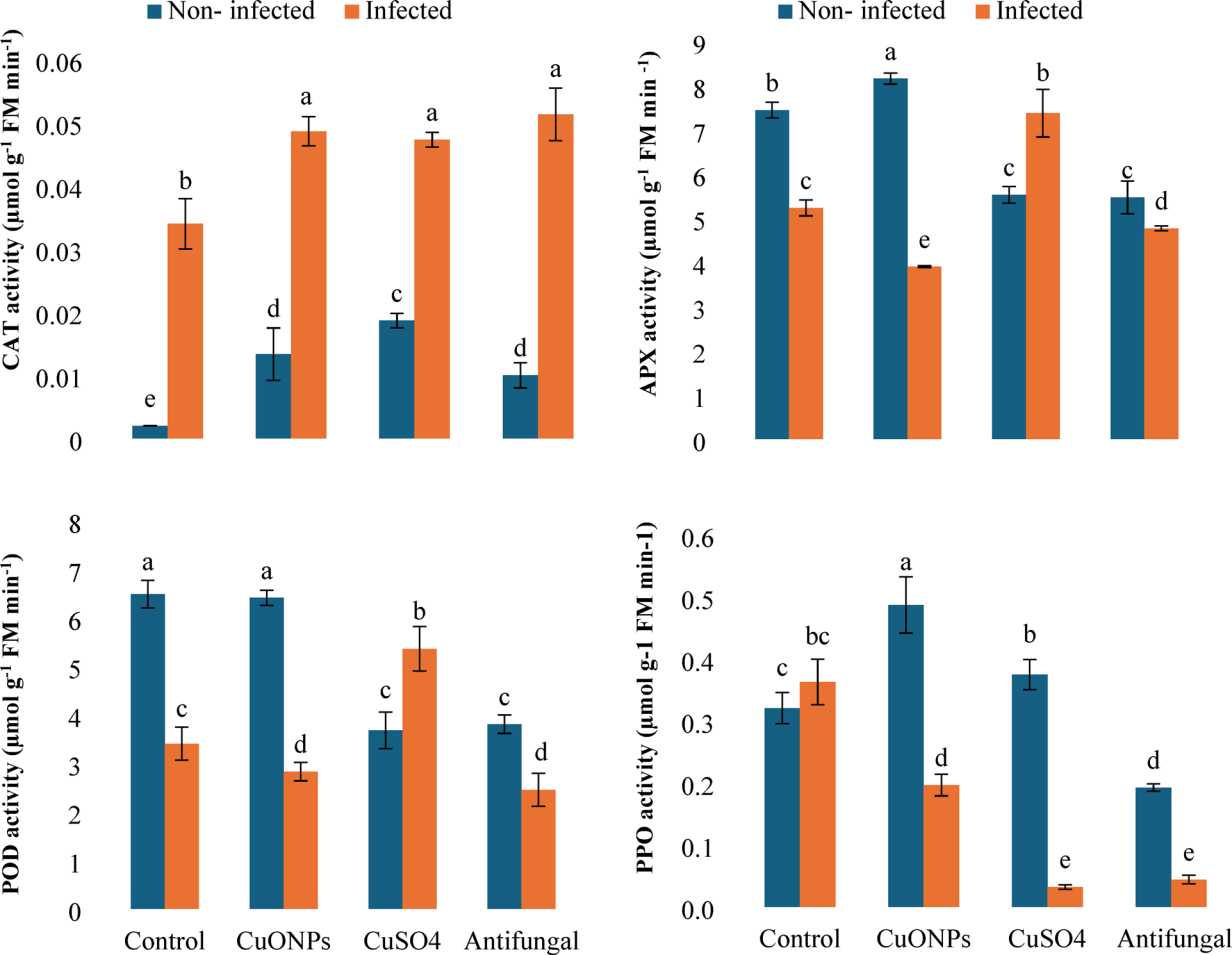
Antioxidant enzyme (CAT, APX, POD, and PPO) activities in *F. falciforme*-infected potato plants following foliar application of myco-synthesized CuONPs (200 mg L^− 1^). Data are presented as mean ± SD (*n* = 3). Different lowercase letters indicate statistically significant differences (*P* < 0.05) according to one-way ANOVA followed by Tukey HSD post-hoc test.

Foliar treatments utilized in this experiment elicited distinct and stress-contextual modulation of the potato antioxidant enzyme system (Fig. 7). *Fusarium falciforme* infection alone moderately induced polyphenol oxidase (PPO, + 11.6%) but suppressed ascorbate peroxidase (APX, −29.7%) compared to healthy controls. Notably, catalase (CAT) activity, which was undetectable in healthy untreated plants, was strongly induced by all treatments, with the most pronounced increases observed in infected plants treated with the commercial antifungal (+ 50.0%), followed by myco-synthesized CuONPs (+ 44.1%) and bulk CuSO_4_ (+ 38.2%).

The enzymatic response to CuONPs was characterized by a balanced, moderate induction. In infected plants, CuONPs significantly elevated CAT while causing a moderate reduction in APX (−25.4%) and PPO (−46.0%), suggesting a substantial shift in the primary detoxification pathway. In contrast, bulk CuSO_4_ acted as a significant inducer of peroxidases under pathogen stress, significantly stimulating both APX (+ 40.9%) and POX (+ 57.3%) activities, while severely suppressing PPO (−91.2%). This indicates a strong pro-oxidant or stress-amplifying effect from ionic copper. The commercial antifungal generally suppressed enzyme activities across both plant health statuses, particularly APX and PPO. The modulation of antioxidant enzymes by CuONPs, particularly the induction of CAT, contributes to their biopesticide action by mitigating oxidative stress induced by pathogen infection.

#### Potato yield

**Fig. 8:**
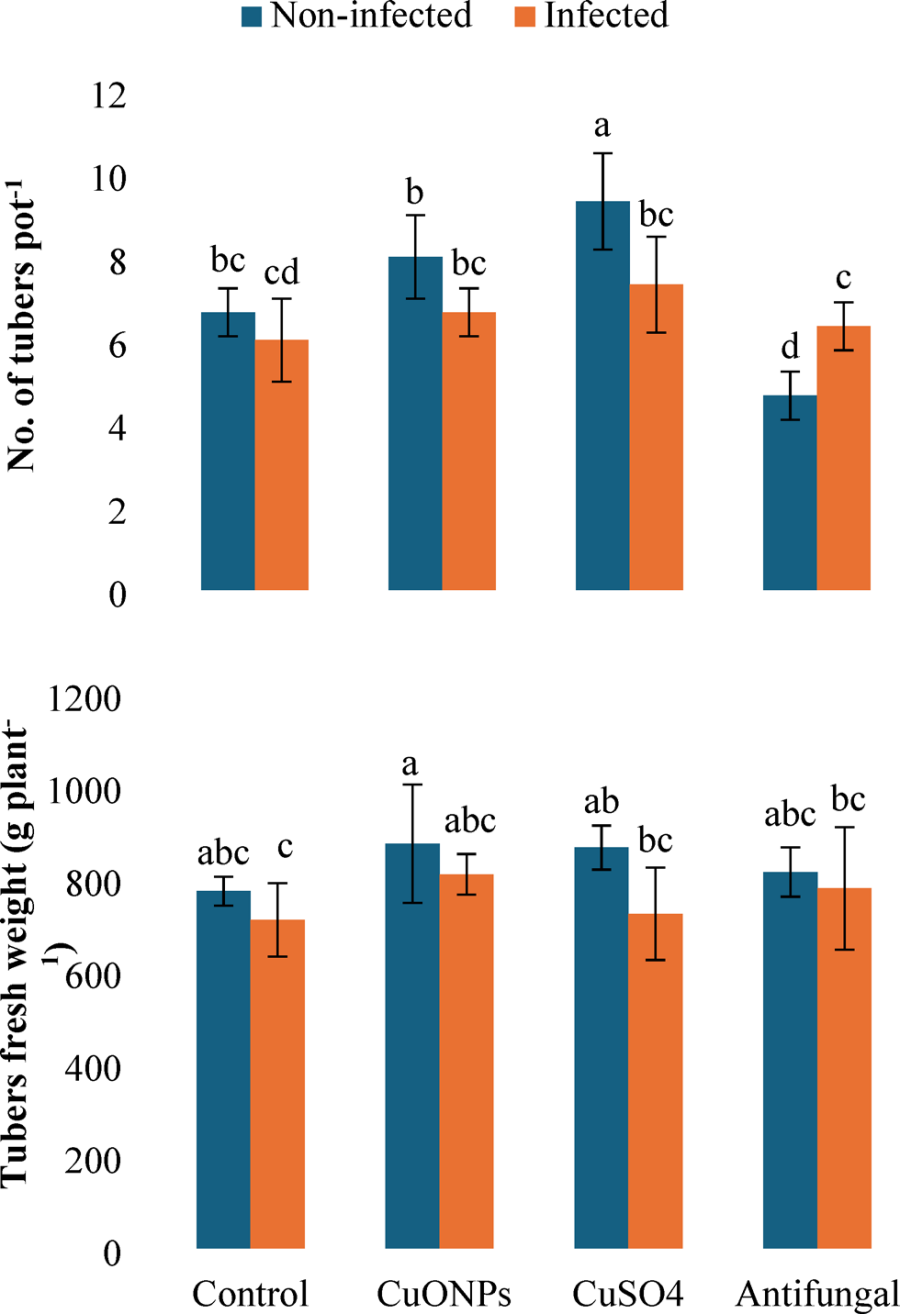
Yield parameters in *F. falciforme*-infected potato plants following foliar application of myco-synthesized CuONPs (200 mg L^− 1^). Data are presented as mean ± SD (*n* = 3). Different lowercase letters indicate statistically significant differences (*P* < 0.05) according to one-way ANOVA followed Tukey HSD post-hoc test.

The effect of the foliar application of myco-synthesized CuONPs, bulk CuSO_4_, and the commercial antifungal Maxim treatments significantly influenced the final tuber yield, with effects modulated by the plant’s infection status (Fig. 8). *F. falciforme* infection individual treatment reduced tuber number and fresh weight per pot of potato by 10.0 and 8.0%, respectively, compared to the healthy controls. Myco-synthesized CuONPs demonstrated a potent growth-promoting effect, particularly in non-infected plants, where they increased tuber number by 20.0% and fresh weight by 40.0%. In infected plants, CuONPs not only mitigated the pathogen-induced yield loss but also enhanced tuber number and fresh weight by 11.1 and 13.8%, respectively, relative to the infected control, restoring productivity to levels surpassing the healthy control. Bulk CuSO_4_ also improved yield in both conditions, though its effect was more moderate. The commercial antifungal exhibited a contrasting outcome, where it increased fresh weight in all plants but significantly decreased tuber number (−30.0%) in healthy plants, indicating a potential phytotoxic effect on reproductive development. These results underscore the dual benefit of myco-synthesized CuONPs as both a biofertilizer (growth-promoting effect) and as a biopesticide (disease-mitigating and yield-restoring effect). Therefore, CuONPs can be classified as a dual-action nano-agrochemical with both biofertilizer and biopesticide properties.

## Discussion

The observed reduction in shoot length, leaf area, and chlorophyll a in *Fusarium falciforme*-infected potato plants aligns with a classic pathogenic response, where biotic stress disrupts host physiology to facilitate colonization^40^. The concomitant, slight induction of chlorophyll b and carotenoids may represent an initial, compensatory plant defense, a transient upregulation of accessory pigments to maintain photosynthetic efficiency under stress, as noted in early infection stages^41^. However, this response is insufficient to prevent overall growth inhibition, consistent with findings in other *Fusarium*-host pathosystems^42,43^.

The foliar application of myco-synthesized CuONPs (200 mg L^− 1^) elicited a notably restorative and growth-promoting effect. By mitigating the pathogen-induced decline in shoot length and leaf area, as well as distinctively reversing the reduction in chlorophyll a, CuONPs demonstrated an ability to preserve both the structural and functional integrity of the photosynthetic apparatus. This protective action likely stems from a multi-faceted mechanism: (1) direct antifungal activity reducing pathogen load and associated toxin production^44^, and (2) nano-specific properties that enhance nutrient availability, potentially acting as a micro-nutrient source or stimulating metabolic pathways^45,46^. The superior performance of CuONPs over bulk CuSO_4_ is critical; while ionic copper can be phytotoxic, inhibiting shoot elongation and inducing oxidative stress at comparable concentrations^47^, the nano-formulation appears to deliver copper in a more bioavailable, less toxic form, thereby separating its beneficial from its deleterious effects.

The commercial fungicide (Maxim) also improved growth and pigment metrics, particularly in infected plants, supporting its primary role in pathogen suppression. However, its efficacy was context-dependent and, in some parameters, less consistent than nano-treatment. The divergent response to bulk CuSO_4_, exhibiting clear phytotoxicity in healthy plants (reduced shoot length) and failing to protect leaf area in infected ones, highlights a fundamental advantage of the nano-engineering approach. It underscores that the benefits of CuONPs are not merely due to copper ions but are intrinsically linked to their nanoscale characteristics, which modulate uptake, translocation, and bioactivity, thereby converting a potential stressor into a potent plant protectant and growth stimulant.

The significant accumulation of carbohydrates concurrently with a sharp decline in protein content in *F. falciforme*-infected potato plants delineates a classic metabolic shift towards osmoprotection under biotic stress. The 52.6% increase in carbohydrates likely represents a compensatory osmotic adjustment, mobilizing sugars to maintain cellular turgor and potentially fuel defense-related biosynthetic pathways^48^. Conversely, the severe 45.9% depletion of protein indicates substantial proteolysis, likely triggered by pathogen-derived effectors and reactive oxygen species, which compromise nitrogen assimilation and redirect resources from growth to survival^49^.

The application of myco-synthesized CuONPs appeared to modulate this dysregulated metabolic state in infected plants. By substantially restoring protein content (+ 26.4%) while only slightly elevating carbohydrates (+ 2.9%), CuONPs appear to mitigate the pathogen-induced protein degradation. This suggests a role in protecting the plant’s nitrogen economy, possibly by reducing oxidative damage to proteins or suppressing protease activity induced by the fungus. In healthy plants, the same treatment induced carbohydrate accumulation (+ 31.0%) but reduced protein levels (−30.2%), indicating a distinct, context-dependent metabolic influence where CuONPs may prioritize carbon storage in the absence of a pathogen attack. This dual functionality aligns with findings where copper nanomaterials improved overall plant vigor and metabolite profiles^49^. The mechanism may involve enhanced micronutrient uptake and the catalytic role of copper in key enzymes of carbohydrate metabolism^45^. In contrast, bulk CuSO_4_ showed a limited ability to restore protein homeostasis (+ 19.6%) and had a negligible effect on carbohydrates (+ 2.0%) in infected plants, underscoring the superior efficacy of the nano-formulation. Furthermore, its protein-reducing effect in healthy plants highlights its inherent phytotoxic potential, a well-documented consequence of ionic copper stress^50,51^.

The commercial fungicide induced the most pronounced carbohydrate accumulation in both plant states (+ 50.6% and + 47.4%) while strongly suppressing protein synthesis, particularly in healthy plants (−52.0%). This pattern suggests that fungicides’ primary action, aggressive pathogen suppression, may inadvertently impose a high metabolic cost, diverting resources towards osmotic solute production (e.g., sugars) at the direct expense of structural and enzymatic proteins, as observed with other systemic fungicides^52,53^. Thus, the myco-synthesized CuONPs distinctively supported a more balanced metabolic outcome under disease stress, favoring the preservation of protein reserves essential for recovery and growth, while the conventional fungicide promoted a carbohydrate-heavy, protein-poor metabolic profile indicative of high defense cost.

Secondary metabolites, particularly phenolics and flavonoids, constitute a cornerstone of the plant’s inducible chemical defense arsenal, with their biosynthesis often upregulated in response to biotic and abiotic stresses to mitigate oxidative damage^54^. The distinct responses observed in this study, where *F. falciforme* infection alone decreased phenolic content (−12.2%) but increased flavonoids (+ 20.3%), underscore the complexity and specificity of phenylpropanoid pathway regulation. This differential modulation suggests a targeted reprogramming rather than an induction, potentially prioritizing the synthesis of specific flavonoid subclasses with enhanced antimicrobial or antioxidant properties over general phenolic accumulation^55^.

Myco-synthesized CuONPs and the commercial fungicide further suppressed both phenolic and flavonoid levels in infected plants. This downregulation may indicate a successful attenuation of the primary oxidative burst triggered by the pathogen, reducing the need for high-level synthesis of these secondary antioxidants. In essence, by directly suppressing the pathogen and associated oxidative stress, CuONPs may alleviate the plant’s need to invest heavily in this particular defensive metabolic route, allowing resource reallocation to other recovery processes^56^. This is consistent with findings where effective nanoparticle treatments reduced phenolic load while enhancing enzymatic antioxidant systems^50^.

In contrast, bulk CuSO_4_ acted as a potent inducer of both phenolics (+ 23.8%) and flavonoids (+ 30.9%) accumulation in infected plants. This pronounced upregulation strongly suggests that ionic copper acts as a secondary abiotic stressor, synergizing with the biotic stress from *F. falciforme* to amplify the oxidative signal. This stress-stacking effect likely overwhelms the plant’s primary detoxification systems, forcing a compensatory overproduction of non-enzymatic antioxidants like phenolics and flavonoids. Therefore, the suppression of phenylpropanoid metabolites by CuONPs in infected plants likely reflects a successful, low-cost defense strategy where the nanoparticle itself mitigates the oxidative challenge. Conversely, their induction by bulk CuSO_4_ signals a high-cost, distress response to combined biotic and abiotic stress. Thus, CuONPs provide protection without imposing the additional metabolic burden associated with ionic metal toxicity, leading to a more efficient and sustainable plant defense outcome.

The final tuber yield represents a decisive, integrative measure of a plant’s physiological and metabolic performance under stress. The reduction in tuber number and fresh weight observed in *F. falciforme*-infected plants confirms the pathogen’s detrimental impact on the plant’s reproductive sink strength and biomass partitioning, a common consequence of vascular wilt diseases that disrupt nutrient and water flow^49,57^. The concurrent increase in tuber dry matter percentage (+ 21.6%) likely reflects stress-induced dehydration or a shift in carbon allocation under compromised water status, rather than a true gain in assimilate partitioning.

The foliar application of myco-synthesized CuONPs demonstrated a notable capacity not only to protect but to enhance yield. In infected plants, CuONPs reversed the pathogen-induced yield loss, increasing both tuber number and fresh weight relative to the diseased control. Crucially, in non-infected plants, CuONPs acted as a potent growth promoter, boosting tuber number by 20.0% and fresh yield by a substantial 40.0%. This dual functionality as a plant growth stimulant and a disease-protective agent underscores a significant agronomic value beyond mere pathogen suppression. This aligns with studies where Cu-based nanoparticles improved fruit set, fresh weight, and overall productivity in various crops, including cucumber, tomato, and onion^46,58,59^. The mechanism likely involves enhanced nutrient use efficiency, improved photosynthetic performance, and the direct role of copper in critical enzymatic processes related to carbohydrate metabolism and lignification. In contrast, bulk CuSO_4_ showed a more moderate yield-enhancing effect, while the commercial fungicide exhibited a critical trade-off, as it increased fresh weight but significantly suppressed tuber yield (−30.0%) in healthy plants. This phytotoxic effect on reproductive development highlights a potential drawback of conventional chemical controls, which can impose metabolic costs that directly compromise yield components in the absence of disease stress.

## Conclusion

This study successfully establishes a green, mycogenic route for synthesizing well-characterized copper oxide nanoparticles (CuONPs) and demonstrates their significant potential in sustainable plant disease management. The myco-synthesized CuONPs exhibited superior antifungal efficacy against *F. falciforme* compared to their bulk counterpart, highlighting the advantage of the nano-formulation. Beyond direct pathogen suppression, the application of CuONPs conferred systemic resilience to potato plants under biotic stress. This was evidenced by the mitigation of growth stunting, the restoration of photosynthetic efficiency, and the modulation of key metabolic and antioxidant pathways, particularly the enhancement of catalase-mediated detoxification. The compelling outcome included a positive impact on yield, as CuONPs acted as a potent plant growth stimulant, increasing tuber production in healthy plants and effectively reversing pathogen-induced yield losses. In contrast, the commercial fungicide exhibited undesirable phytotoxic effects on tuber development in healthy plants. For farmers and policymakers, CuONPs appear to be an eco-friendly alternative to commercial fungicides for both *F. falciforme* control and productivity enhancement. Future research should focus on field-scale validation and elucidating the long-term environmental interactions of these bio-fabricated nanoparticles to entirely recognize their capacity in integrated pest management systems.

## Data Availability

The sequence generated in this study for Fusarium falciforme strain Ff-22 has been deposited in the GenBank database under accession number PZ167595 and is publicly available at: https://www.ncbi.nlm.nih.gov/nuccore/PZ167595.1?report=genbank. The data supporting the findings of this study are available within the article and from the corresponding author (Khalil Saad-Allah) upon reasonable request.
